# Repurposing the estrogen receptor modulator raloxifene to treat SARS-CoV-2 infection

**DOI:** 10.1038/s41418-021-00844-6

**Published:** 2021-08-17

**Authors:** Marcello Allegretti, Maria Candida Cesta, Mara Zippoli, Andrea Beccari, Carmine Talarico, Flavio Mantelli, Enrico M. Bucci, Laura Scorzolini, Emanuele Nicastri

**Affiliations:** 1 Dompé Farmaceutici S.p.A, L’Aquila, Italy; 2 Sbarro Health Research Organization, Biology Department CFT, Temple University, Philadelphia, PA USA; 3 Lazzaro Spallanzani National Institute for Infectious Diseases, IRCCS, Rome, Italy

**Keywords:** Infectious diseases, Drug development

## Abstract

The ongoing coronavirus disease 2019 (COVID-19) pandemic caused by the novel severe acute respiratory syndrome coronavirus 2 (SARS-CoV-2) necessitates strategies to identify prophylactic and therapeutic drug candidates to enter rapid clinical development. This is particularly true, given the uncertainty about the endurance of the immune memory induced by both previous infections or vaccines, and given the fact that the eradication of SARS-CoV-2 might be challenging to reach, given the attack rate of the virus, which would require unusually high protection by a vaccine. Here, we show how raloxifene, a selective estrogen receptor modulator with anti-inflammatory and antiviral properties, emerges as an attractive candidate entering clinical trials to test its efficacy in early-stage treatment COVID-19 patients.

## Facts

1. Despite the recent advancements in vaccines development, it is still essential not to underestimate the risk associated with COVID-19 infection, and to advance knowledge on specific SARS-CoV-2 pharmacological treatments to cure or mitigate the impact of COVID-19 on patients.
2. Drug repurposing is an attractive approach to address unmet clinical needs in infectivology.
3. The E4C program has shown the potential of powerful supercomputing platforms in shortening the timing to the identification of potential clinical candidates targeting key viral proteins leading to the selection of an estrogen receptor (ER) modulator, raloxifene, currently under clinical investigation.
4. Literature data suggest a protective role of estrogens in COVID-19 infection, by regulation of cytokines related to immunity and inflammation, suggesting ER modulation as a promising pharmacological approach for preventing/attenuating the cytokine storm and inflammation associated to COVID-19.

## Open questions

1. Need to identify new therapies addressing noncritical COVID-9 patients.
2. Selective estrogen modulators (SERMs) can modulate activity and replication of SARS-CoV-2 by interacting with estrogen receptors (ERs).
3. If successful, the ongoing clinical study on raloxifene, a well-known SERM, in paucisymptomatic COVID-19 patients could represent a valid opportunity of treatment for controlling disease progression.
4. Validation of the running hypothesis would pave the way for further investigation on ER modulation as therapeutic approach to treat other infectious diseases.

## Introduction

In December 2019, a new identified coronavirus, severe acute respiratory syndrome coronavirus 2 (SARS-CoV-2), caused an outbreak in Wuhan with public health crisis in China and rapid spread across the world, causing general concern. On February 11, 2020, the World Health Organization officially named the disease “COVID-19”; the Chinese Government took strong and harsh measures to control the outbreak progression characterized by atypical pneumonia cases. Similar measures were also adopted in Europe and worldwide. Common symptoms of COVID-19 include fever, sore throat, fatigue, cough, shortness of breath, and dyspnea that may eventually progress toward acute respiratory distress syndrome (ARDS), to the involvement of other systems/organs are also susceptible to COVID-19 (e.g., heart, liver, and kidneys) [1, 2], up to the death in the most critical cases. About 80% of patients have mild-to-moderate disease, 14% have severe disease, and 6% are critical (namely, they develop respiratory failure, septic shock, and/or multiple organ dysfunction/failure) [3].

Coronaviruses are a large family of viruses belonging to the *Coronaviridae* family. The limited number of coronaviruses known to circulate in humans widely cause mild infections, and are relatively harmless respiratory human pathogens. The culprit virus belongs to the family of coronaviruses that caused two other outbreaks, namely SARS in 2002 [4], and Middle East respiratory syndrome (MERS) in 2012 [5]. The first known SARS-CoV case occurred in Foshan, China, in November 2002, and new cases emerged in mainland China in February 2003. The first emergence of MERS-CoV occurred in June 2012 in Saudi Arabia. These events demonstrate that the threats of CoVs must not be underestimated and that it is essential to advance the knowledge on these viruses, their replication, and interactions with the hosts with the main goal to develop new pharmacological treatments and vaccines.

Investigations on epidemiological and clinical characteristics as well as outcomes of patients infected by SARS-CoV-2 demonstrated that the infection causes clusters of severe respiratory illness similar to the known SARS-CoV. The symptoms of human infection with SARS-CoV-2 are generally fever, fatigue, dry cough, and dyspnea. Noteworthy, a considerable percentage of COVID-19 cases rapidly progress to a severe and critical clinical condition, where acute lung injury (ALI), ARDS, and pulmonary edema are the most common complications in a large number of hospitalizations with the need of supplemental oxygen, invasive and noninvasive mechanical ventilation, or even, in very few cases of extra corporeal membrane oxygenation.

In this situation, new strategies to treat and stop the spread of the disease or mitigate the impact of COVID-19 both on patients and the national health system are urgently needed. Specifically, they should aim at: (1) preventing patients with mild COVID-19 to progress toward severe/critical disease, (2) relieving hospitals from the burden of highly complicated patients, and (3) shortening the time of viral shedding in order to curb individual infectiousness and reduce the average number of secondary cases. Although knowledge and understanding of COVID-19 are rapidly evolving, as are supportive medical cares, there is still much uncertainty on the proposed treatments’ real efficacy. This fact is mainly imputable to the accumulation of data from small and uncontrolled trials, observational studies, and case studies, most of them from the initial Chinese epidemic, that are affected by several biases, intense background noise, and a long list confounding factors [6]. In any case, due to the urgent need of effective treatments, several drugs with variable targets have been and continue to be proposed for the treatment of severe or mild-to-moderate COVID-19 patients worldwide, even if systemic corticosteroids treatment and intensive care life-support therapies as of today represent the only effective treatments to reduce mortality for patients with critical COVID-19. A therapy addressed to noncritical conditions may be relevant for the public health care to meet the medical needs of a high number of patients requiring specific therapeutic support. Several antivirals have been proposed as potential effective therapies against COVID-19, some of them tested due to their earlier beneficial role against other coronaviruses such as MERS-CoV and SARS-CoV. In contrast, others have shown favorable outcomes in preclinical in vitro studies, among them several anti- human immunodeficiency virus (HIV) protease inhibitors including lopinavir–ritonavir [7], darunavir–cobicistat [8–10], a broad-spectrum inhibitor of viral RNA polymerase, favipiravir [11], and remdesivir [12]. In particular, remdesivir has been authorized by the FDA for emergency use in the treatment of suspected or laboratory-confirmed COVID-19 in adults and children hospitalized and with severe disease (https://www.fda.gov/media/137564/download), and in last July 2020 was approved by EMA under 1-year conditional marketing authorization (https://www.ema.europa.eu/en/news/first-covid-19-treatment-recommended-eu-authorisation). However, the proven antiviral efficacy of these drugs in COVID-19 patients is generally limited by their low bioavailability in the lungs [13], and their clinical efficacy is still controversial due to limited randomized efficacy clinical trials [14], so much co that no conclusive results are available today [15].

## Drug repurposing approach

The identification and development of effective drugs in new indications are lengthy processes that are not adequate to face the emergency of the immediate global challenge generated by the COVID-19 outbreak. Hence, in the present unprecedented situation of deaths daily due to the epidemic, repurposing existing drugs with proven safety and toxicology profiles leverage huge advantage in terms of sparing time in identifying new potential treatments [16, 17]. Drug repurposing provides a particularly attractive approach to address unmet clinical needs in the area of infectious diseases. This “recycling” strategy based on the reuse of approved drugs is mostly successful, as demonstrated by examples of repurposing treatments in cancer and other human diseases [18]. Drug repurposing is a particularly attractive approach that substantially reduces time and costs of drug development, as safety, tolerability, and pharmacokinetic data already exist [19]. For this reason, the pandemic emergency immediately triggered large efforts to identify potential effective treatments within the pharmaceutical armamentarium for the management of severe/critical patients and to contain the spread of COVID-19 infection. Several clinical trials and drug repositioning studies have been conducted and many studies are currently ongoing to select new agents to control this pandemic better. Drugs with broad and different mechanisms of action have been selected as potential candidates for the treatment of COVID-19 patients, such as antiviral, immune enhancing, anti-inflammatory, or immunomodulatory agents [20]. Among the drugs previously approved for different indications and currently tested in ongoing or completed clinical trials against COVID-19, several very different compounds and biologicals exist. Worth mentioning are chloroquine and hydroxychloroquine to interfere with the viral entry and replication, anti-interleukin-6 receptor monoclonal antibodies (anti-IL-6R) for reducing the inflammatory response upon cytokine storm [21], and corticosteroids, previously used to treat SARS and MERS infections, and frequently administered to COVID-19 patients to reduce lung inflammation and prevent ARDS development [22]. Table 1 reports a comprehensive, but not exhaustive, list of the most popular repurposed drugs approved or under development for COVID-19 treatment.

**Table 1:** List of the most popular repurposed drugs approved or under development for COVID-19 treatment.

Broad mechanism of action	Drug name	Status
Antiviral therapies	Remdesivir	Approved (FDA)^a^ EMA conditional marketing authorisation (EMA)^b^
	Favipiravir	Approved (CDSCO)
	Lopinavir/Ritonavir ± ribavirin	Phase 4 [8]
	Ribavirin	Phase 2 [87]
	Oseltamivir	Phase 3 [88]
	Chloroquine/Hydroxychloroquine	Phase 4 [89]
Promoters of the innate antiviral response	Antiviral Immune suppression (type-I IFN-α, type-I IFN-β, type-III IFN-λ)	Phase 2 [90] Phase 2 [91] Phase 2 [92]
	Exogenous interferon therapy	Phase 2 [93]
	Azithromycin	Phase 4 [94]
Immunomodulatory and anti-inflammatory drugs	Immunosuppressive therapies	Phase 3 [95]
	Tocilizumab	Phase 3 [96]
	Siltuximab	Phase 3 [97]
	Sarilumab	Phase 3 [98]
	Anakinra	Phase 3 [99]
	Corticosteroids	Approved^c^ [100]
Antihistamine	Famotidine	Phase 3 [101]
Analgesic, antiemetic	Icatibant	Phase 2/3 [102]
Respiratory stimulant	Almitrine	Phase 3
Protease inhibitor	Darunavir	Phase 3 [103]
Janus kinase inhibitor	Ruxolitinib	Phase 2 [104]
Miscellaneous drugs	Ivermectin	Phase 2 [105]

^a^https://www.fda.gov/drugs/news-events-human-drugs/remdesivir-veklury-approval-treatment-covid-19-evidence-safety-and-efficacy.

^b^https://www.ema.europa.eu/en/medicines/human/EPAR/veklury.

^c^https://www.covid19treatmentguidelines.nih.gov/therapies/immunomodulators/corticosteroids/.

## Gender sensitivity in COVID-19 and SARS-CoV-2

A gender disparity in COVID-19 severe cases and case fatality rate have been reported in China, where the infection rate among males and females was similar, but the death rate among males was 4.7% compared with 2.8% for females. Likewise, in Italy, a higher death rate in male patients (14.8%) than in female patients (8.2%) was reported by the Italian National Institute of Health, and similar trends have been reported in the USA, China [23], and South Korea [24]. Several factors, genetic, hormonal, and behavioral can contribute to the observed gender disparity, although experimental data are lacking so far. Adopting a sex and gender-informed perspective in research has already improved patient care in other therapeutic areas affecting both women and men [25]. It is well known that male and female subjects respond differently to many viral infections, or pathogens in general, due to a more intense and more vigorous immune response modulation [26], either innate or adaptive, and that in female subjects the viral clearance of pathogens is favored [27] as well as a greater vaccine efficacy [28] and vaccine adverse events [29].

On the contrary, testosterone has mainly suppressive effects on immune function, which could partially explain the greater susceptibility to infectious diseases observed in men. In agreement with this, the reduction of testosterone in ageing men has been associated with increased levels of proinflammatory cytokines, known to contribute to the worsening of COVID-19 progression in older patients [30]. Sex differences in disease progression could also be linked to estrogen-induced reduced expression of the protein ACE2, a validated receptor for SARS-CoV-2 entry into the target cells [31]. A recent paper reports the results of a cross-sectional study of COVID-19 patients in China [32] that demonstrates that females have a better prognosis than males, nonmenopausal women have a shorter length of hospital stays, and anti-Müllerian hormone and estradiol are negatively correlated with COVID-19’s severity. Further, there was a negative correlation between estradiol and the levels of IL-6, IL-8, IL-2R, and tumor necrosis factor α (TNF-α), which are significantly correlated with disease severity or composite endpoints. Very recently, a paper was published to support the above hypotheses [29] further. The authors’ findings highlight a positive and protective effect of estrogen from symptomatic COVID-19, based on the positive association between predicted COVID-19 and menopausal status and the negative association of contraceptive pill use.

## Role of estrogens in gender sensitivity to COVID-19 and SARS-CoV-2

It is noteworthy that estrogens are known to modulate the expression of ACE2, the functional receptor for both SARS-CoV [33] and SARS-CoV-2 [34]. Most of the literature reports an upregulation of ACE2 by estrogens but there is also some evidence of a possible downregulation by 17β-estradiol (E2) [35, 36]. This different behavior could be correlated with complex genetic, X-chromosome related effects, including ACE2 different regulation. These interactions would eventually improve the outcome of the COVID-19 infection by reducing inflammation and the cytokine storm, thrombosis, fibrosis, and modulating the immune response, thus inhibiting the increased capillary permeability, coagulation, fibrosis, and apoptosis in the alveolar cells and ensuing accelerated lung damage [37].

Interestingly, previous literature (both in animals and humans) highlighted sex-specific differences in susceptibility to SARS-CoV and perhaps other coronavirus infections. Experimental studies in male and female mice infected with SARS-CoV showed that male mice have a higher susceptibility to SARS-CoV infection and higher mortality than females, consistently with the human disease. Ovariectomy in female mice or treatment with estrogen antagonists increased females’ death rate, which could be rescued by treatment with agents belonging to the class of selective estrogen receptor (ER) modulators (SERMs). Enhanced susceptibility of male mice to SARS-CoV was associated with elevated virus titer, enhanced vascular leakage and alveolar edema. These changes were accompanied by increased accumulation of inflammatory monocyte macrophages (IMMs) and neutrophils in male mice’s lungs; depletion of IMMs partially protected mice from lethal SARS [38]. Taken together, these data seemed to support that sex bias does exist in response to coronavirus infection, probably in some extent due to the protective role of nonmenopause and sex hormones, especially E2, by regulation of cytokines related to immunity and inflammation. This and other observations opened doors to large number of studies aimed at understanding the relation between hormonal balance and infection susceptibility, as well as infectious diseases progression and prognosis.

The above experiments in animal models emphasized a protective role of estrogens in infection, protective action of SERMs, and the effect of estrogen hormones was also analyzed in other contexts not limited to Coronaviruses. For example, mice treated with exogenous estriol (E3) during infection with mouse-adapted H1N1 influenza virus had reduced total pulmonary inflammation and improved disease outcomes compared with females that received no hormone [39]. In an in vitro model of hepatitis C infection, 17β-estradiol inhibited hepatitis C virus (HCV) life cycle by interfering with viral assembly/release [40].

**Fig. 1:**
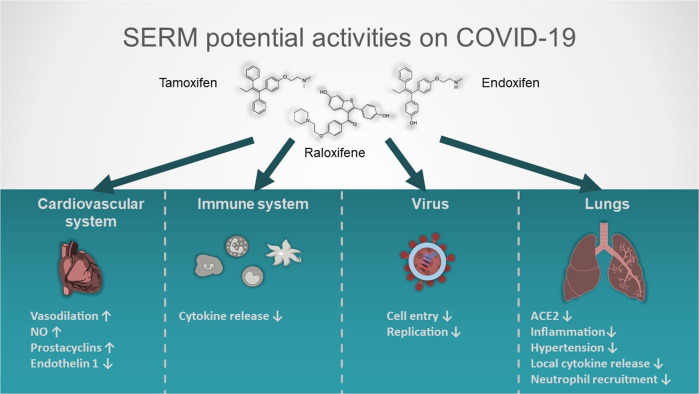
SERM potential activities on COVID-19. Effect of different SERM on organs, viruses and immune system. The up arrow means positive modulation; the down arrow negative modulation.

From a mechanistic point of view, clinical and experimental studies strongly suggest that estrogens can modulate lung inflammation and allergic reactions, because ER activation modulates immune cells, both innate and adaptive immune responses, and downregulate lung inflammation and its capacity of releasing cytokines [41, 42]. Estrogens also attenuate the vasoconstrictor response to various stimuli and induce vasodilation in the pulmonary vasculature during stress situations [43]. This event is mediated by increased levels of prostacyclin and nitric oxide (NO) and decreased levels of endothelin-1. All these mechanisms play an essential role in improving outcomes in the setting of ALI and other critical conditions such as trauma, shock, sepsis and myocardial ischemia/reperfusion. The beneficial effects of estrogens on immune response mechanisms, viral infection, lung inflammation, and the cardiovascular system have been also highlighted as relevant factors influencing the disease progression and outcome [44] (Fig. 1).

Based on this rationale, clinical trials are currently ongoing or are ready to start, to evaluate the effect of estrogen treatment of COVID-19. A phase 2 study has been started to test if E2 delivered via a transdermal patch can reduce the severity of COVID-19 symptoms compared to regular care in COVID-19 positive or presumptive positive patients (NCT04359329). In contrast, another trial (NCT04539626) will have the objective to evaluate the effect of additional estradiol estrogen therapy on clinical response and mortality in nonsevere COVID-19 patients. Finally, a clinical trial will investigate the change in men’s clinical status with COVID-19 hospitalized and treated with oral progesterone (NCT04365127).

## Selective ER modulators

**Fig. 2:**
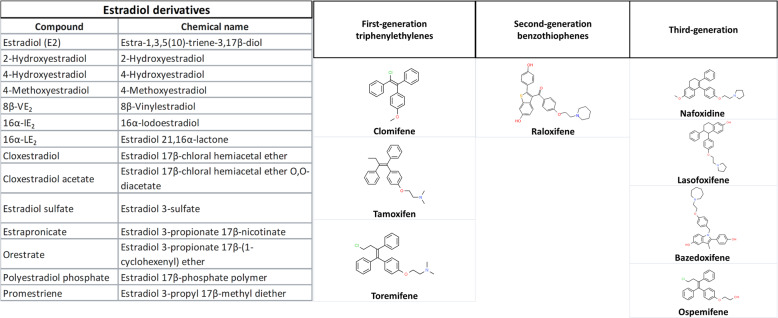
17β-estradiol derivatives and different SERM generations.

**Fig. 3:**
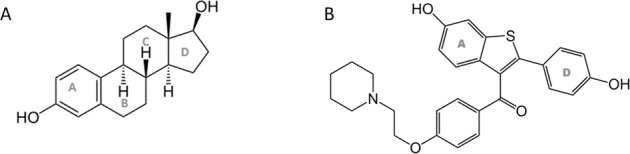
Structural analogies between SERMs and steroids. **A** The ABCD steroid ring system in 17β-estradiol. **B** “A ring” and “D ring” marked in raloxifene.

**Fig. 4:**
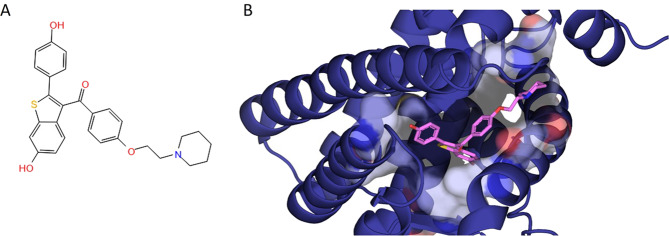
Binding mode of raloxifene to ERα. **A** 2D chemical structure of raloxifene and **B** three-dimensional schematic representation of raloxifene (RAL) complexed with the Estrogen Receptor (ER) Alpha Ligand Binding Domain (PDB Code: 7KBS). RAL and ER are reported in pink sticks and blue cartoon, respectively. The binding site region is shown in transparent surface.

SERMs are a group of nonsteroidal compounds acting as ligands for ERs and have the unique feature to behave as agonists or antagonists in a target gene and in a tissue-specific fashion [45, 46]. Approximately 70 molecules with a SERM-like pharmacological profile are known; SERMs approved for clinical use mainly belong to the chemical classes of triphenylethylene derivatives, benzothiophenes, napthylenes, indoles, and benzopyrans (Fig. 2). The structure–activity relationships of this class have been deeply studied leading to a progressive improvement of the potency and selectivity profile in the newest SERM generations [47]. Binding mode of SERM to ER has been widely characterized together with the chemical and structural analogies with steroid ligands (Fig. 3) which account for the observed biological activities. In particular, raloxifene, such as 4-hydroxytamoxifen, binds to ERα with the hydroxyl group of its phenolic “A ring” through hydrogen bonds with Arg-394 and Glu-353. In addition to these bonds, raloxifene forms a second hydrogen bond to ER through the side group of His-524 because of the presence of a second hydroxyl group in the “D ring” [48] (Fig. 4). One of the key pharmacological characteristics of SERMs lies in their mixed agonism/antagonism profile that is responsible of beneficial estrogenic effects in specific target tissues, at the same time avoiding adverse or off-target effects. ERs have a large and flexible binding pocket that accommodates multiple ligands, such as steroids, phytoestrogens, and other molecules. The ligand–ER binding causes a receptor conformational change that promotes receptor dimerization and activation, thus causing either the direct binding to the estrogen response element or the binding to coregulator proteins at their own promoter site. The two ERs, α and β, have three main domains, the N-terminal domain, the DNA binding doman, and the ligand binding domain, that have specific roles and contain activation function domains whose activation occurs in a tissue-dependent and promoter-dependent manner [49–51], and is responsible for recruiting transcriptional coregulator proteins after receptor stimulation. These proteins are able to interact with ERs, thus modulating gene transcription, and highlighting the relevance of selective activation of the regulatory proteins based on both ER subtype and ligand binding activity. The development of new SERM generations has progressively made possible new therapeutic applications of estrogen-related therapy. To date, the main indications are prevention and treatment of breast cancer, prevention of osteoporosis, and maintenance of beneficial serum lipid profile in postmenopausal women, even some related side effects, such as thromboembolic events and carcinogenesis, have emerged for some of them hindering their development, especially in case of long-term treatments. Thus, new SERM generations are structurally distinct from the previous classes and show the ability on one hand to show a strong antiestrogenic effect on breast and endometrial tumors, and on the other hand able to stimulate the formation of new bone via a mechanism estrogen dependent [45].

## SERM antiviral effect

An overexpression of ER is considered to play a crucial role in inhibiting viral replication [52]. SERMs have been reported to play a broader role in inhibiting viral replication through the nonclassical pathways associated with ER. SERMs interfere at the postviral entry step and affect the triggering of fusion, as the “SERMs” antiviral activity still can be observed in the absence of detectable ER expression. In line with the above, several preclinical and clinical studies show a broad-spectrum antiviral action of SERMs, among them raloxifene [53]. The studies exploring the antiviral activity of SERMs were mainly focused on three infections: HIV, HCV, and Ebola virus (EBOV). Tamoxifen, first-generation SERM, was found active against HIV, HCV, and herpes simplex virus 1 [54]. In in vitro studies, tamoxifen showed the ability to block the upregulation of HIV-1 replication induced by phorbol myristate acetate (a protein kinase C activator) in CD4+ T lymphocytes [55], and to inhibit HCV genome replication by abrogating the HCV RNA polymerase NS5B association with the replication complex which is functionally regulated by the ER alpha [56]. Also toremifene, belonging to the same chemical class as tamoxifen and marketed for the treatment of oncological indications, was deeply investigated for its potential antiviral activity in MERS-CoV and SARS-CoV [57–59]. The antiviral effectiveness of SERMs was further confirmed in models of EBOV infection where these compounds showed the capacity to delay the viral infection through cell-based mechanisms unrelated to the classic estrogen signaling pathways [53, 60]. Further, in coronaviruses, cell lines toremifene prevents fusion between the viral and endosomal membrane by interacting with and destabilizing the viral membrane glycoprotein, also affecting several key host proteins associated with human coronavirus [61]. Taking together the main findings so far available on direct and indirect mechanisms underlying the effects of estrogens as adjuvants for the treatment of SARS-CoV-2 infection, SERMs may be regarded as multitargeted drugs able to interfere with several processes, including virus entry and replication [44]. Table 2 shows antiviral activity of relevant SERMs. Interestingly, recent data [62] on the prevalence of SARS-CoV-2 infection, hospitalization, and death in oncological patients in treatment with SERMs shed light on possible mechanisms and protective effects due to SERMs, thus suggesting new and possible ways to prevent or mitigate the effects of the virus.

**Table 2:** Selective estrogen receptor modulators (SERM) and in vitro antiviral activity.

SERM	Virus	IC_50_ (μM)	IC_50_ (μM) Exscalate
Raloxifene	SARS-CoV-2	0.020^a^	3.74
	Ebola virus	1.24 ± 0.5 [106]	
	HCV	1 [52]	
Tamoxifen	SARS-CoV-2		5.95
	MERS-CoV	10.12 [107]	
	Ebola virus	0.75 [108]	
Toremifene	SARS-CoV	11.97 [108]	
	MERS-CoV	12.91 [57]	
	SARS-CoV-2	4.77–11.3 [109]	6.4
	Ebola virus	0.0016 + 0.048 [53]	
Clomiphene	SARS-CoV-2	5.36 [110]	13.62
	Ebola virus	1.83 [108]	

^a^CN111728973.

## Raloxifene: pharmacological activity and safety profile

Raloxifene is the generic name of 1-[6-hydroxy-2-(4-hydroxyphenyl)benzo[b]thien-3-yl]-1-[4-[2-(1-piperidinyl)ethoxy]phenyl]methanone [63]. This drug is a second-generation, selective, benzothiophene SERM with agonist or antagonist activity on estrogen-responsive tissues. Like E2, raloxifene crosses the cytoplasmic membrane and the nuclear membrane; in the cell nucleus, the benzothiophene ring binds to the ER with a comparable affinity to E2. Raloxifene is the active ingredient of branded (Optruma^®^—Eli Lilly or Evista^®^—Daiichi Sankyo) or generic drugs (Raloxifene Teva) marketed as 60 mg oral tablets. The drug is registered in Europe since 1998 for the treatment and prevention of osteoporosis in postmenopausal women, and in the USA since 1997 for the treatment and prevention of osteoporosis, and reduction of the risk of invasive breast cancer in postmenopausal women. The drug has a double receptor activity, either as an estrogen agonist in some tissues (bone, lipid metabolism) or as an estrogen antagonist in others, such as endometrium and breast, producing some of the estrogen’s beneficial effects with low occurrence and severity of adverse events [64, 65]. In bone and in other nonreproductive tissues, raloxifene bound to ER activates a specific sequence of DNA known as the raloxifene responding element, a group of genes that regulates the synthesis of specific cell proteins responsible for the estrogen agonist effect. Raloxifene decreases bone resorption and overall bone turnover, thus increasing bone density with little or no effect on the endometrium, resulting in no predisposition to uterine cancer. The drug is also able to lower total cholesterol LDL in the serum, with no effect on HDL [66]. This can be regarded as beneficial for cardiovascular prevention; nonetheless, thrombotic events and related cardiovascular events attributed to the treatment with raloxifene occasionally occurred. Raloxifene also exerts an anti-inflammatory effect via a decreased production of IL-6 (up to 50%) and decreased production of TNF-α (up to 30%) [67]. In terms of safety profile, it is important to keep in mind that raloxifene has been in use for more than 20 years to treat and prevent osteoporosis in postmenopausal women. The recommended dose is 60 mg/day and is intended for long term (chronic) use. This means more than 20 years in post-marketing exposure, in addition to extensive clinical studies supporting the approved indication and other conditions. The clinically most important adverse reactions reported are venous thromboembolic events, together with more common and less relevant adverse reactions (≥1/100) such as headache, hot flushes, nausea, vomiting, abdominal pain, dyspepsia, rash, leg cramps, mild breast symptoms, flu syndrome, peripheral edema, and increased blood pressure. Raloxifene safety profile is supported by a huge volume of clinical data from long-term treatments mostly focused on the cardiovascular and thromboembolic risks. More than 7700 postmenopausal women with osteoporosis were exposed to raloxifene in the MORE Study [68] with a 3-year follow-up period; as the main outcome, raloxifene therapy for 8 years did not significantly affect the risk of cardiovascular events in patients. Similarly, in the RUTH study in which a total of 10,101 women were followed-up for a median of 5.6 years, raloxifene did not significantly affect the risk of CHD compared to placebo [69]. In the STAR trial, almost 20,000 postmenopausal women at increased risk of invasive breast cancer were treated with either oral tamoxifen or raloxifene over 5 years, showing lower thromboembolic events in the raloxifene group (RR: 0.70; 95% CI: 0.54–0.91) [70]. Further, even if not authorized for use, the drug is well tolerated in men, resulting from a randomized, blinded study vs placebo, including 50 year older adults. The treatment with 60 mg/day raloxifene for 6 months was well tolerated, and no severe adverse reactions occurred; the only significant difference in adverse effects was a higher occurrence of hot flushes [71]. Given at the dose of 120 mg/day for 6 months to healthy male subjects, raloxifene increases levels of LH, FSH, and total testosterone [72]. The safety profile was also assessed in special populations of patients (renal impairment, hepatic dysfunction). All the results were enclosed in the European SmPC (https://www.ema.europa.eu/en/documents/product-information/evista-epar-product-information_en.pdf). Table 3 summarizes adverse reactions and their frequencies observed in treatment and prevention studies involving over 13,000 postmenopausal women along with adverse reactions arising from postmarketing reports. The duration of treatment in these studies ranged from 6 to 60 months. The majority of the adverse events did not require discontinuation of therapy. Regarding possible safety issues attributable to the use of higher doses of raloxifene, up to 600 mg/day was effectively used in past clinical studies, no significant difference between active treatment groups was observed in a dose-ranging clinical study with raloxifene 200 and 600 mg. Comparable efficacy was observed with only a greater frequency of hot flushes, at the higher dose (600 mg) (EPAR, https://www.ema.europa.eu/en/documents/scientific-discussion/evista-epar-scientific-discussion_en.pdf). Bearing in mind that each SERM presents a unique risk/benefit profile based on varying indications and tissue-specific ER agonist and antagonist effects, the long-term safety profile of SERMs is largely acceptable and, among them, raloxifene, designed for long-term treatments such as osteoporosis, has a good safety profile, one of the best within the SERM class, running in parallel with a pharmacological profile that indicates a good risk/benefit ratio and tolerability, while aware of the possible side effects [73–75].

**Table 3:** Summary of adverse reactions and frequencies observed with raloxifene.

Adverse drug reactions^a^	Frequency	Disorder
Blood and lymphatic system disorders	Uncommon	Thrombocytopenia
Nervous system disorders	Uncommon	Fatal stroke
	Common	Headache, including migraine
Vascular disorders	Very common	Vasodilatation (hot flushes)
	Uncommon	Venous thromboembolic events, including deep vein thrombosis, pulmonary embolism, retinal vein thrombosis, superficial vein thrombophlebitis, and arterial thromboembolic reactions
Gastrointestinal disorders	Very common	Symptoms such as nausea, vomiting, abdominal pain, and dyspepsia
Skin and subcutaneous tissue disorders	Common	Rash
Musculoskeletal and connective tissue disorders	Common	Leg cramps
Reproductive system and breast disorders	Common	Mild breast symptoms a such as pain, enlargement, and tenderness
General disorders and administration site conditions	Very common	Flu syndrome
	Common	Peripheral edema
Investigations	Very common	Increased blood pressure

^a^Term(s) included are based on postmarketing experience for raloxifene (http://www.ema.europa.eu; Annex I—Summary of product Characteristics—Evista^®^ 60 mg). The following convention has been used to classify the frequency of adverse reactions: very common (≥1/10), common (≥1/100 to <1/10), uncommon (>1/1000 to <1/100), rare (>1/10,000 to <1/1000), and very rare (<1/10,000).

## Raloxifene: a promising SERM for the treatment of SARS-Cov2 infection

Raloxifene has been found to have an in vitro antiviral activity, in terms of inhibition of viral replication and/or infection, against EBOV [54, 76, 77], HCV [78, 79], HBV [80], and Zika virus [81]. Further, it showed efficacy in human female cells from nasal epithelium, against the influenza virus A [82]. Raloxifene was found efficacious in a randomized, clinical study (raloxifene plus standard of care (SOC) vs SOC only) in postmenopausal women [83] as an adjuvant antiviral treatment of chronic hepatitis C (CHC), by improving the efficacy of SOC for the hepatitis C treatment with pegylated interferon α2a plus ribavirin. The sustained virological response rate was significantly higher for raloxifene plus SOC patients (61.3%) than for SOC only patients (34.4%) (*p* = 0.0051) after 24 weeks of treatment. These results in patients with CHC suggest that raloxifene is a promising candidate as an adjuvant in the antiviral treatment of CHC patients. Hypotheses have been made on the antiviral mechanism of action of raloxifene which are based on an effect on the entry of the enveloped virus at an early stage of infection (effect on autophagy and endocytosis), and on a viral envelope–host membrane interaction with effect on phospolipidosis (inhibition of lysosomal lipid metabolism and vesicular trafficking), modulation of cholesterol metabolism (reduction of cholesterol content in the viral membrane that destabilizes the same), and increase of NO levels that leads to suppression of the viral replication cycle [84]. Recently, several molecules with potential efficacy against SARS-CoV-2 were selected using the EXaSCale smArt pLatform Against paThogEns (EnsEXSCALATE) supercomputing platform (a powerful tool for the repurposing of drugs and compounds in new indications), the core of a Horizon 2020 European Project “N.101003551-EXSCALATE4CoV” (call: H2020-SC1-PHE-CORONAVIRUS-2020) (E4C) granted to counteract the Coronavirus pandemic and improve the management and care of patients. The project involves a public–private consortium headed by Dompé Farmaceutici S.p.A.

**Fig. 5:**
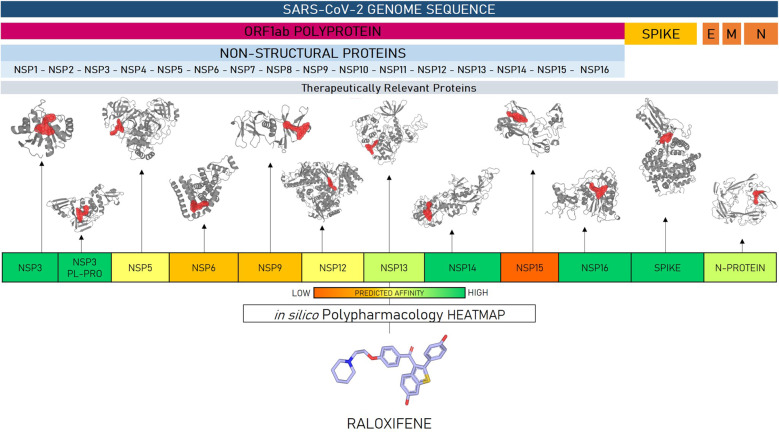
In silico polypharmacological effects of raloxifene performed on the main SARS-CoV-2 proteins. In supplementary information a predicted structure complex with raloxifene is reported for each SARS-CoV-2 protein.

The EXSCALATE platform is currently one of the most powerful and cost-efficient intelligent supercomputing platforms at global level, able to screen huge “chemical libraries” of 500 billion compounds, and process more than 3 million molecules per second, thus able to predict and identify the best molecules addressing a selected target, and enabling them to further characterization. The combination of advanced CADD techniques with high throughput biochemical and phenotypic screening allows a rapid evaluation and shortening of new drugs’ discovery timing. This approach has proved extremely useful in pandemic infections by viruses and other pathogens, where immediate response and the swift identification of effective treatments are of paramount importance. In this context, E4C brought to select raloxifene as a clinical candidate against SARS-CoV-2 by using an integrated approach between the EXSCALATE platform and the virtual screening protocols, really supportive in predicting the high probability of the drug to interact with several relevant SARS-CoV-2 targets (Fig. 5). Among the molecules identified with potential antiviral activity, raloxifene was selected as a promising molecule to treat mild-to-moderate symptoms, and paucisymptomatic COVID-19 patients due to the predicted ability to bind (Fig. 5 and Supplementary information) relevant SARS-CoV-2 proteins such as the PLpro protease and the spike protein (open data from https://opendata.ncats.nih.gov/covid19/assays). In line with previous studies on SARS and MERS viruses, together with raloxifene, other members of the SERM class were predicted from EXSCALATE in silico studies as potential candidates, including tamoxifen, clomiphene, and toremifene, as confirmed by other independent computational studies [85]. Both literature and our experimental in vitro data confirmed raloxifene as the most potent molecule of the SERM class acting at low concentrations (micromolar range) against SARS-CoV-2 and other CoVs (Table 2). The drug potency is higher than other popular antivirals such as remdesivir, lopinavir, and chloroquine, further confirming the potential for clinical development. Additional drug characteristics support the clinical use like a favorable pharmacokinetic profile. In fact, raloxifene, while showing a low oral bioavailability (less than 2%), has higher pulmonary distribution than other compounds of the class. Raloxifene exerts its pharmacological activity at very low circulating levels (nM) but with wide distribution into body tissues, thus reaching appropriate levels mainly in lungs, kidneys, and liver [86], and supporting a direct effect in target tissues in COVID-19 patients. The observation on the efficacy of raloxifene in clinical studies in other antiviral infections further support the prioritization of raloxifene vs other members of the SERM class for a clinical investigation of its potential role in the protection against SARS-CoV-2 infection.

The good distribution profile of raloxifene in the lungs, highly susceptible to viral infections, is particularly relevant since the low concentrations reached in pulmonary tissue is considered one of the major limitations of other antiviral drugs, such as remdesivir, currently used to treat COVID-19 respiratory complications. From a safety point of view, as discussed above, effects due to raloxifene treatment have been extensively studied in postmarketing settings for 22 years in postmenopausal women, resulting in a positive safety profile in the authorized conditions for use. The drug has also been studied in men, and no additional safety risks were checked. The comparison of raloxifene safety profile with other SERMs at the authorized dosage and regimen is an additional driver for the drug selection for clinical studies. Although the safety profile of raloxifene in the treatment of COVID-19 is not yet known, and careful monitoring of potential cardiovascular and thromboembolic risks is mandatory, the short treatment, the dosage, and the target population (paucisymptomatic patients) are relevant to mitigate potential risks. Nevertheless, the design of the clinical study and the patient recruitment criteria introduced specific measures to avoid possible additional risks, especially in terms of cardiovascular and thromboembolic side effects

## Conclusions

The massive information available on small molecules with various levels of potential to act as inhibitors against SARS-CoV-2 possess challenges of its own but, leveraging the advanced technologies like artificial intelligence, machine learning, and high throughput screenings do have the potential to speed up the discovery of new treatments to a great extent. In pandemic situations like present, it is mandatory to create networks of collaborations worldwide to access different approaches with the common goal to solve the problem. The hope is that COVID-19 may subside in relatively few months, as happened with SARS and MERS infections. Nevertheless, the current pandemic has reemphasized the importance of developing new and safe antiviral agents to fight present and future viruses, among them and very important, coronaviruses.

Raloxifene has been selected as a clinical candidate for clinical studies in paucisymptomatic COVID-19 patients based on a scientific rationale and literature evidence supporting both a potential antiviral action and protective action of SERMs through ER-dependent and independent mechanisms, as well as a protective role of the ER-signaling pathway, probably relevant in the observed gender sensitivity to COVID-19 and SARS-Cov-2, as well as to other coronaviruses. Studies to clarify the antiviral mechanism of action and the potential therapeutic role of raloxifene are ongoing. During the European EXSCALATE4CoV project activities, raloxifene was selected through an integrated approach of drug repurposing and in silico screening on SARS-CoV-2 target’s proteins subsequent in vitro screening of several selected compounds. Raloxifene showed an optimal activity profile to advance it further as a drug with a potential COVID-19 indication. The rationale to speed up the development of raloxifene is further supported by its safety profile derived from long-term treatment data, also in special populations; the potential risks of severe side effects, intrinsic to the class of SERMs, are considered negligible for short-term treatments as those planned for future clinical trials in COVID-19 patients. In addition, in in vitro studies, no significant plasma protein binding interactions were observed with warfarin and other compounds such as testosterone. Further, no clinically relevant effects on raloxifene plasma concentrations have been checked so much due to frequently coadministered drugs such as paracetamol, nonsteroidal anti-inflammatory drugs, antibiotics, H1 or H2 antagonists, and benzodiazepine so that the use of combinations of raloxifene with these drugs is allowed in clinical protocols. Summarizing the above data suggests that ER modulation may be a suitable pharmacological approach for preventing/attenuating the cytokine storm and inflammation associated with COVID-19, and in particular the use of SERMs, specifically raloxifene, may represent a promising pharmacological option. Such a therapeutic approach would be beneficial for the treatment of both male and female patients in the early phase with mild/moderate symptoms of the disease, in order to prevent or mitigate the possible evolution toward more severe and dangerous forms of the disease, due to the onset of the cytokine storm, and potentially reducing the viral transmission.

### Search strategy and selection criteria

A large-scale semiautomatic bibliographic search of the literature was performed using the Scopus/Elsevier database, which led to the identification of 10,904 documents containing information referable to raloxifene. Among these documents a restriction was made by downloading in groups of 500 all the documents accessible in full text for a total of 8721 documents. The database thus obtained was annotated with an ad hoc script to identify the names of human genes/proteins (from the UniProt and HGNC databases). Over 600 documents were finally analyzed. Priority was given to prospective studies and reviews with adequate methodological quality. For the analysis of all relevant publications, consensus meetings were held with all the authors.

## Supplementary information

Supplementary Information
