# Supplementary material for: Repurposing the estrogen receptor modulator raloxifene to treat SARS-CoV-2 infection
Source: Cell Death Differ. 2021 Aug 17;29(1):156–66. doi: 10.1038/s41418-021-00844-6 (PMC8370058; doi:10.1038/s41418-021-00844-6)
Supplement: Supplementary file 1 — Supplementary Information [file 41418_2021_844_MOESM1_ESM.docx]

**Supplementary Information**

**(Manuscript CDD-21-0178: “Repurposing the estrogen receptor modulator raloxifene to treat SARS-CoV-2 infection”)**

Supplementary Figures from 1 to 12 show the binding site details of the Raloxifene-viral protein complexes at the atomistic level. The images refer to the Figure 5 of the paper that showing the Raloxifene’s *in silico* polypharmacology.


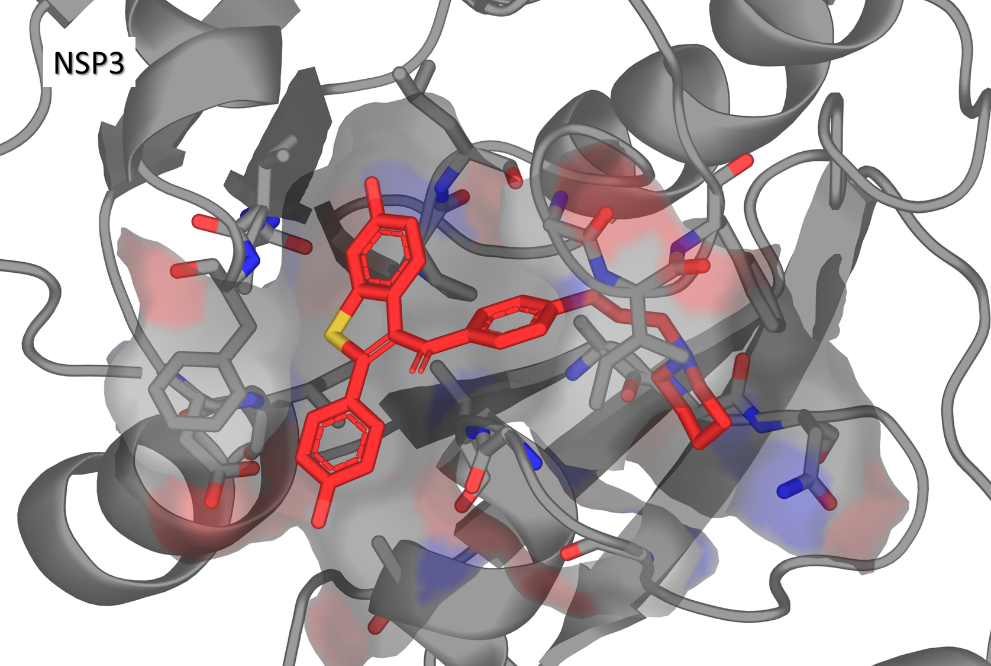


SUPPL. FIGURE 1. Proposed binding mode of Raloxifene on NSP3. The protein is reported in grey cartoon and surface, Raloxifene and key binding site residues are reported in red and gray sticks respectively.


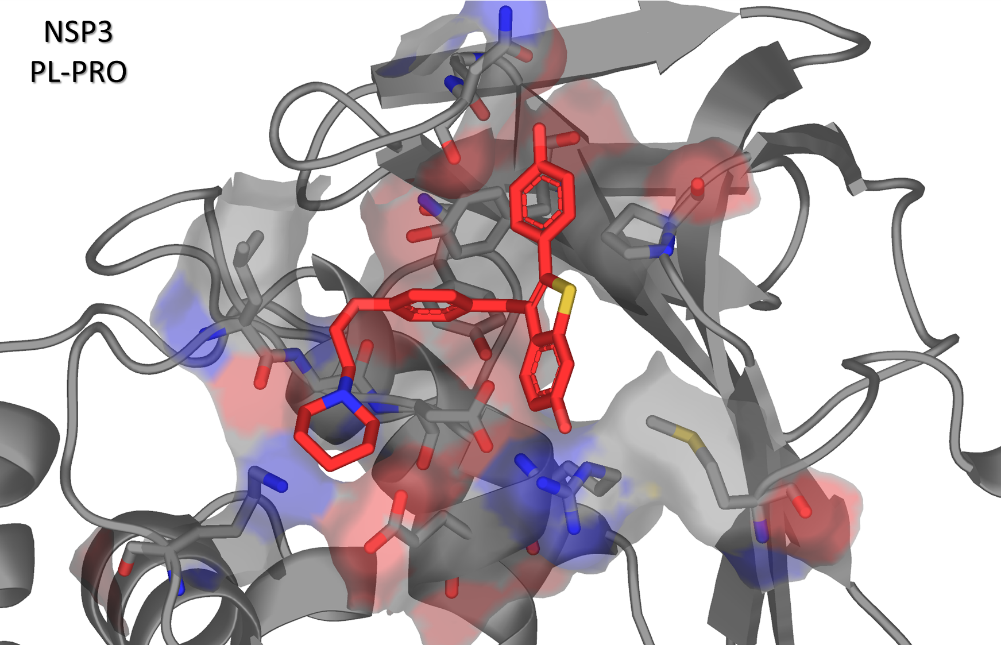


SUPPL. FIGURE 2. Proposed binding mode of Raloxifene on NSP3 PL-PRO. The protein is reported in grey cartoon and surface, Raloxifene and key binding site residues are reported in red and gray sticks respectively.


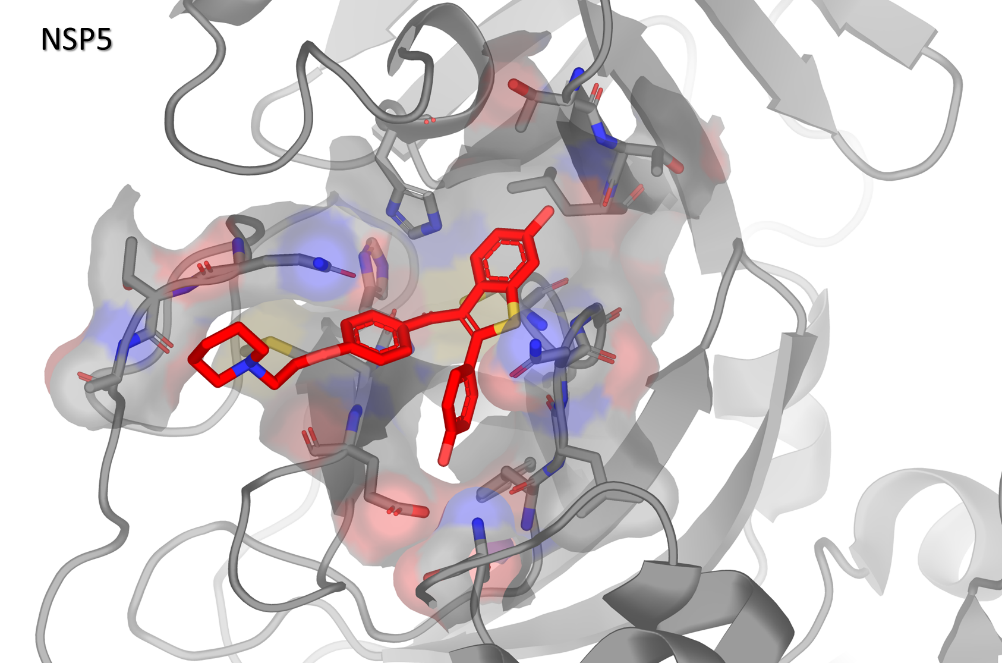


SUPPL. FIGURE 3. Proposed binding mode of Raloxifene on NSP5. The protein is reported in grey cartoon and surface, Raloxifene and key binding site residues are reported in red and gray sticks respectively.


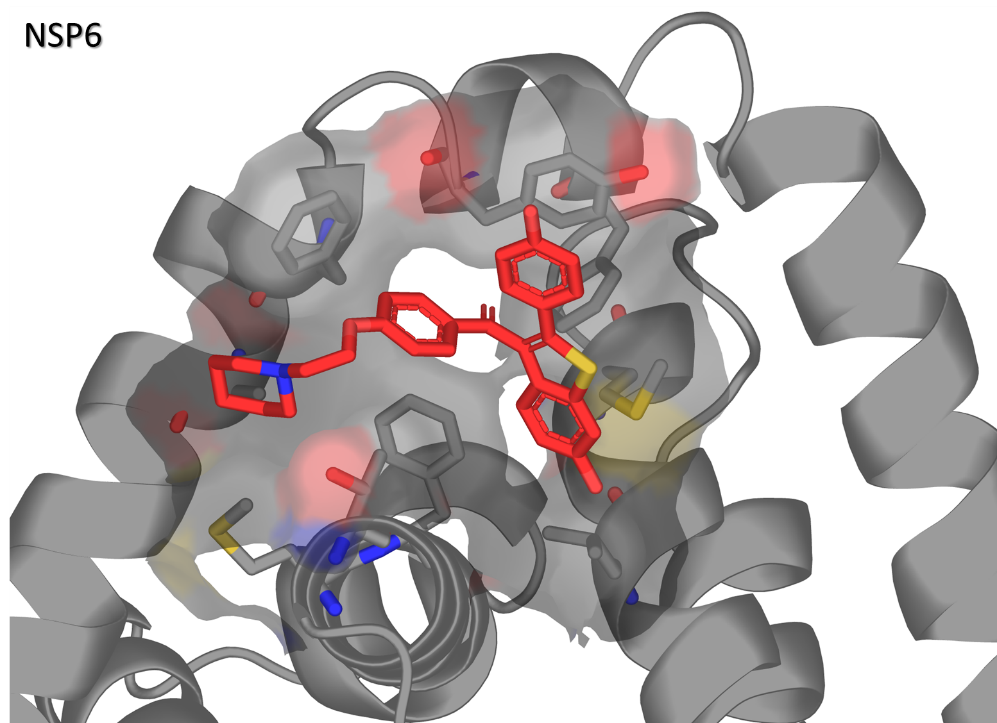


NSP6

SUPPL. FIGURE 4. Proposed binding mode of Raloxifene on NSP6. The protein is reported in grey cartoon and surface, Raloxifene and key binding site residues are reported in red and gray sticks respectively.


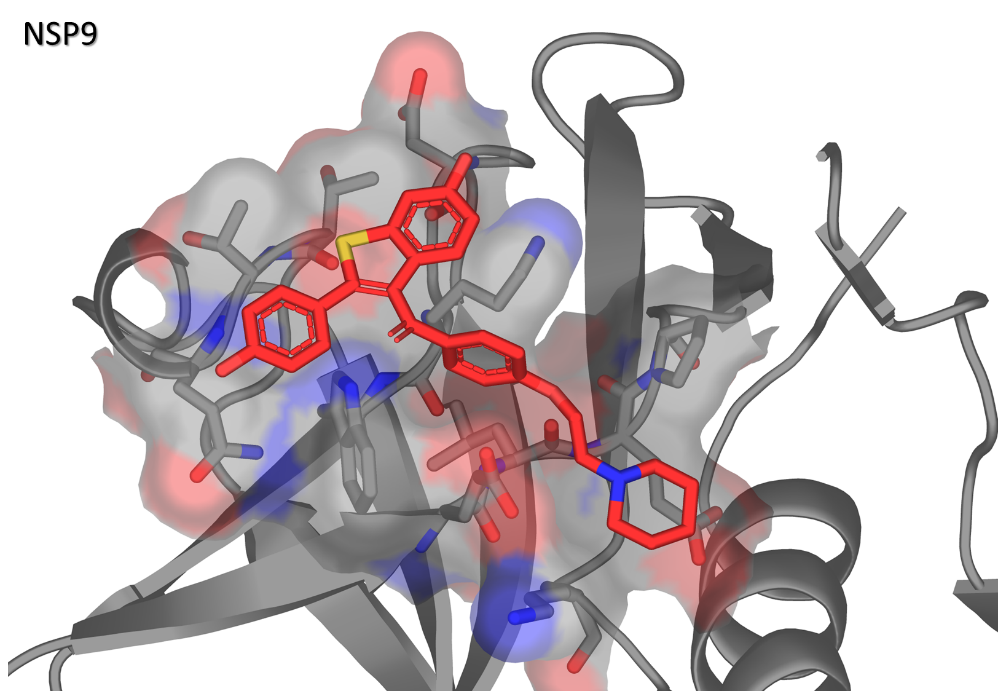


SUPPL. FIGURE 5. Proposed binding mode of Raloxifene on NSP9. The protein is reported in grey cartoon and surface, Raloxifene and key binding site residues are reported in red and gray sticks respectively.


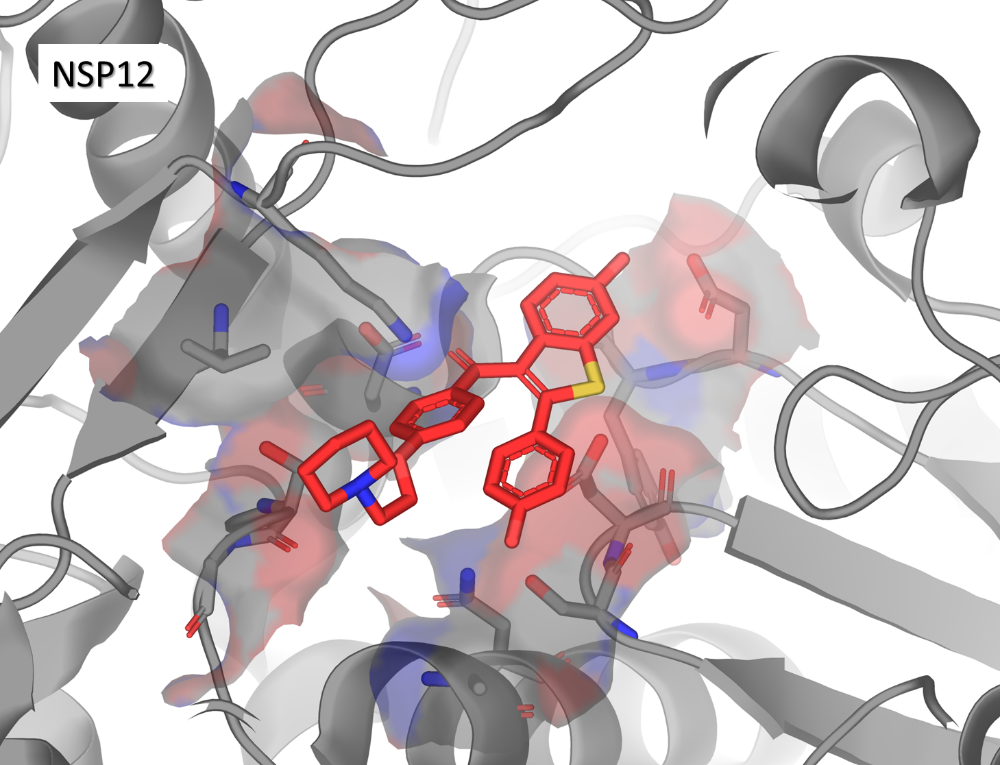


SUPPL. FIGURE 6. Proposed binding mode of Raloxifene on NSP12. The protein is reported in grey cartoon and surface, Raloxifene and key binding site residues are reported in red and gray sticks respectively.


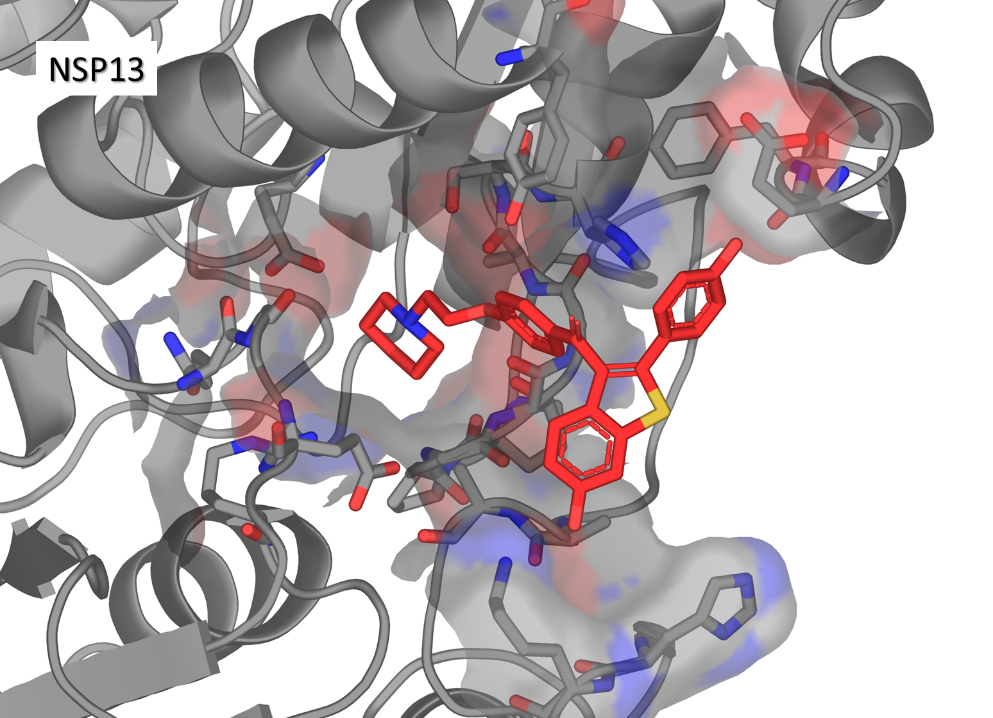


SUPPL. FIGURE 7. Proposed binding mode of Raloxifene on NSP13. The protein is reported in grey cartoon and surface, Raloxifene and key binding site residues are reported in red and gray sticks respectively.


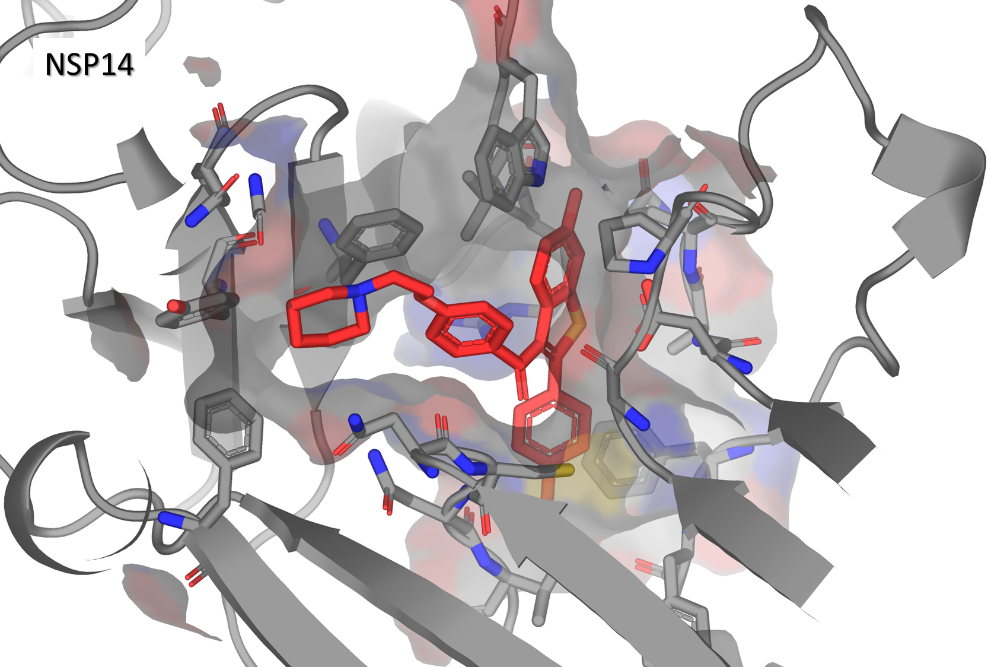


SUPPL. FIGURE 8. Proposed binding mode of Raloxifene on NSP14. The protein is reported in grey cartoon and surface, Raloxifene and key binding site residues are reported in red and gray sticks respectively.


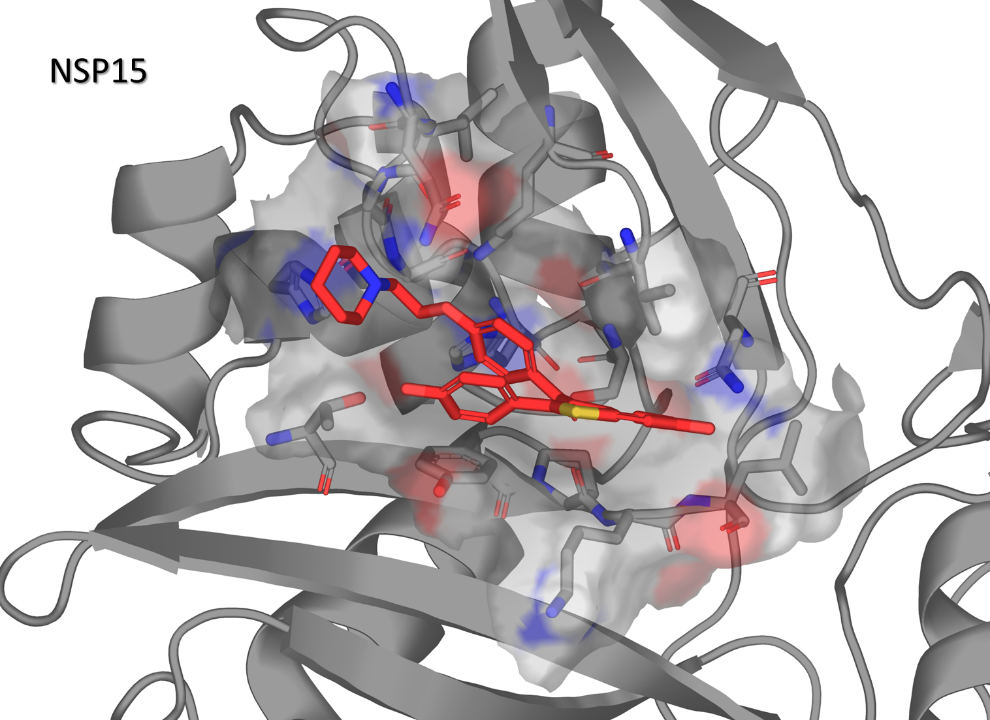


SUPPL. FIGURE 9. Proposed binding mode of Raloxifene on NSP15. The protein is reported in grey cartoon and surface, Raloxifene and key binding site residues are reported in red and gray sticks respectively.


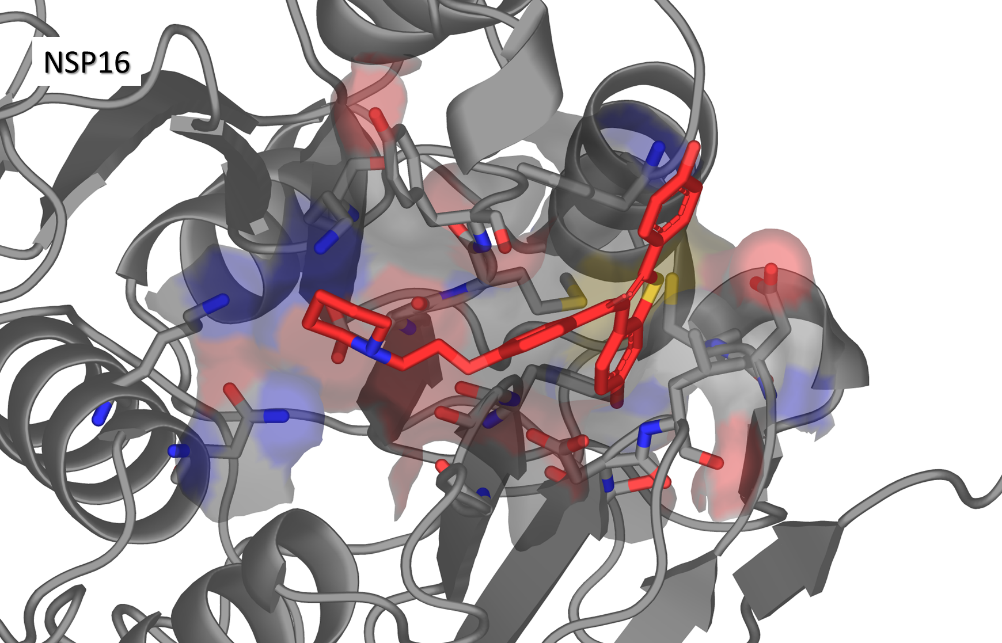


SUPPL. FIGURE 10. Proposed binding mode of Raloxifene on NSP16. The protein is reported in grey cartoon and surface, Raloxifene and key binding site residues are reported in red and gray sticks respectively.


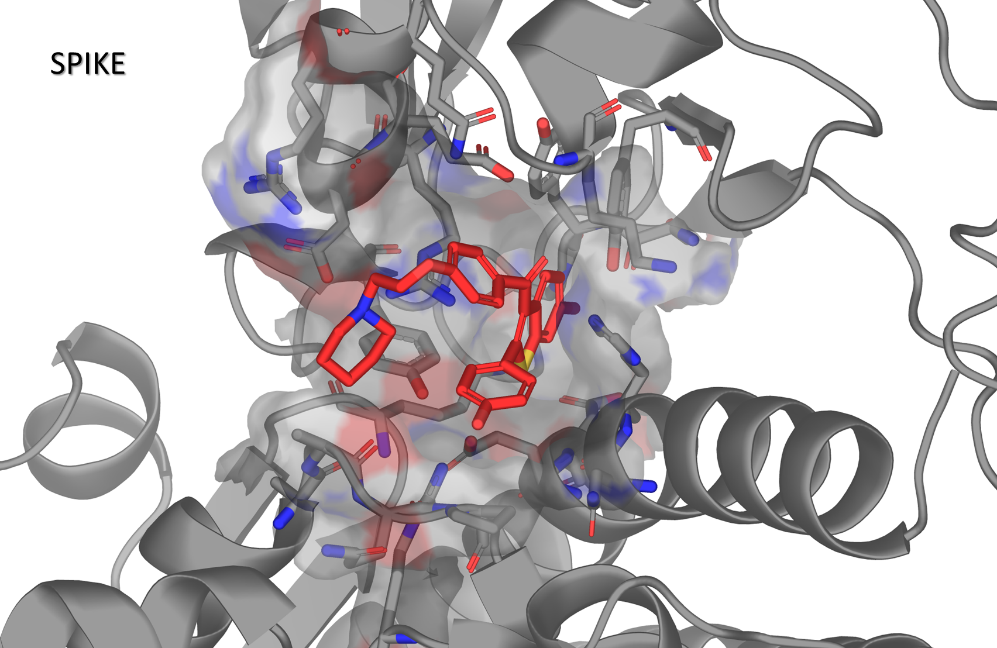


SUPPL. FIGURE 11. Proposed binding mode of Raloxifene on SPIKE. The protein is reported in grey cartoon and surface, Raloxifene and key binding site residues are reported in red and gray sticks respectively.


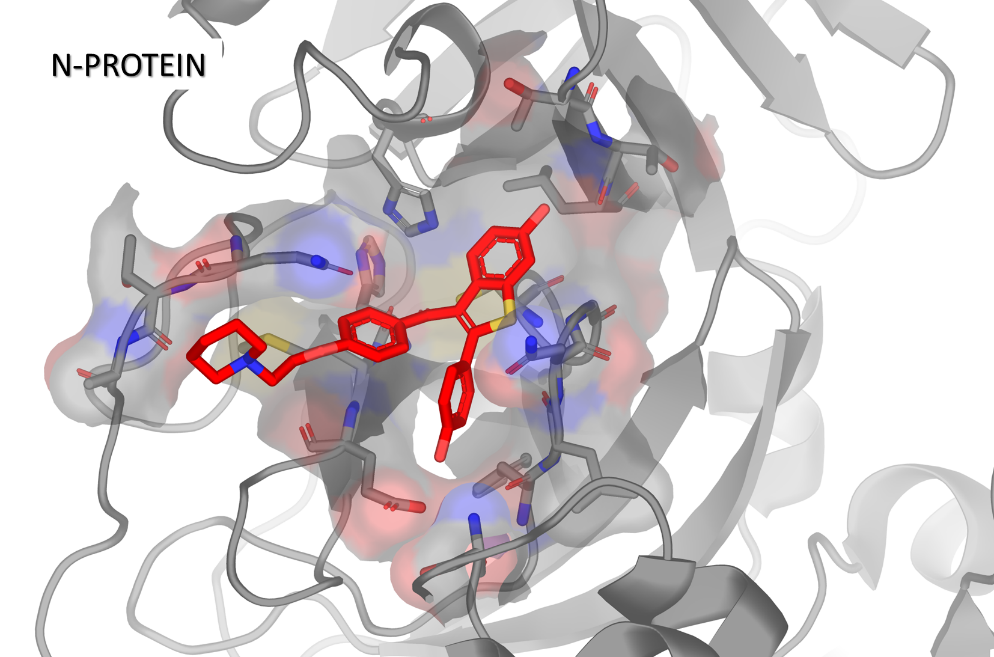


SUPPL. FIGURE 12. Proposed binding mode of Raloxifene on N-PROTEIN. The protein is reported in grey cartoon and surface, Raloxifene and key binding site residues are reported in red and gray sticks respectively.
